# Smelling Nano Aerial Vehicle for Gas Source Localization and Mapping

**DOI:** 10.3390/s19030478

**Published:** 2019-01-24

**Authors:** Javier Burgués, Victor Hernández, Achim J. Lilienthal, Santiago Marco

**Affiliations:** 1 Institute for Bioengineering of Catalonia (IBEC), The Barcelona Institute of Science and Technology, Baldiri Reixac 10-12, 08028 Barcelona, Spain; smarco@ibecbarcelona.eu; 2 Department of Electronics and Biomedical Engineering, Universitat de Barcelona, Marti i Franqués 1, 08028 Barcelona, Spain; 3 AASS Mobile Robot Olfaction Lab, Örebro University, SE 70182 Örebro, Sweden; victor.hernandez@oru.se (V.H.); achim@lilienthals.de (A.J.L.)

**Keywords:** robotics, signal processing, electronics, gas source localization, gas distribution mapping, gas sensors, drone, UAV, MOX sensor, quadcopter

## Abstract

This paper describes the development and validation of the currently smallest aerial platform with olfaction capabilities. The developed Smelling Nano Aerial Vehicle (SNAV) is based on a lightweight commercial nano-quadcopter (27 g) equipped with a custom gas sensing board that can host up to two in situ metal oxide semiconductor (MOX) gas sensors. Due to its small form-factor, the SNAV is not a hazard for humans, enabling its use in public areas or inside buildings. It can autonomously carry out gas sensing missions of hazardous environments inaccessible to terrestrial robots and bigger drones, for example searching for victims and hazardous gas leaks inside pockets that form within the wreckage of collapsed buildings in the aftermath of an earthquake or explosion. The first contribution of this work is assessing the impact of the nano-propellers on the MOX sensor signals at different distances to a gas source. A second contribution is adapting the ‘bout’ detection algorithm, proposed by Schmuker et al. (2016) to extract specific features from the derivative of the MOX sensor response, for real-time operation. The third and main contribution is the experimental validation of the SNAV for gas source localization (GSL) and mapping in a large indoor environment (160 m^2^) with a gas source placed in challenging positions for the drone, for example hidden in the ceiling of the room or inside a power outlet box. Two GSL strategies are compared, one based on the instantaneous gas sensor response and the other one based on the bout frequency. From the measurements collected (in motion) along a predefined sweeping path we built (in less than 3 min) a 3D map of the gas distribution and identified the most likely source location. Using the bout frequency yielded on average a higher localization accuracy than using the instantaneous gas sensor response (1.38 m versus 2.05 m error), however accurate tuning of an additional parameter (the noise threshold) is required in the former case. The main conclusion of this paper is that a nano-drone has the potential to perform gas sensing tasks in complex environments.

## 1. Introduction

**Figure 1:**
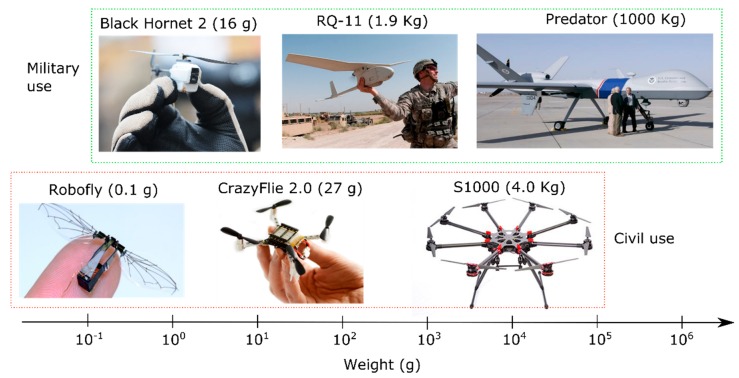
Overview of the UAV landscape, from insect-sized drones to military aircrafts, classified according to the approximate weight and size. The graphic shows the large range of UAV sizes, which spans seven orders of magnitude.

Thanks to recent advances in micro-technology, manufacturers of unmanned aerial vehicles (UAVs), or drones, have been able to develop miniaturized flying platforms; with insect-sized aircrafts expected in the future [1] (Figure 1). A micro-UAV (MAV or μUAV) has a length between 15 cm and 100 cm and a weight between 50 g and 2 kg [2]. A nano air vehicle (NAV) or nano-drone is extremely small, with a wingspan lower than 15 cm, and weighs less than 50 g [2]. If compared to piloted aircrafts or larger UAVs, MAVs and NAVs can fly at low altitudes (i.e., below 150–200 m) over small geographic or site-specific areas on a real-time basis at affordable operational costs [3]. MAVs equipped with gas detection systems and/or sampling bags have been already used in the fields of environmental monitoring [4,5,6,7,8,9], volcanic gas sampling [10,11,12,13,14], localization of fugitive emissions [15,16], early fire detection [17,18], precision agriculture [19,20,21], landfill monitoring [22,23,24], disaster response [25] and mine blasting [26], among others [27,28].

The tiny form-factor and maneuverability of NAVs allow sensing of hazardous environments inaccessible to terrestrial robots and bigger drones, can fly over areas being unobserved and are not a hazard for humans, enabling their use in public areas or inside buildings. Providing a NAV with olfaction capabilities is now possible due to miniaturization and low-cost fabrication of gas sensors. An odor-sensitive nano-drone can be used in a myriad of applications that range from environmental monitoring to search and rescue, leak detection, chemical, biological, radiological and nuclear (CBRN) defense, explosive finding, among others. For example, in the aftermath of an earthquake or explosion it is important to search for victims and hazardous gas leaks inside pockets that form within the wreckage of collapsed buildings. A nano-drone could navigate such scenarios much faster than a terrestrial robot, passing through confined spaces that preclude human entry, evading obstacles or large gaps and sampling the space in three dimensions (3D).

### 1.1. Related Work on Gas-Sensitive Nanodrones

Two experimental works [29,30] already explored the viability of nano-drones for gas sensing tasks. Rossi et al. [29] performed preliminary indoor experiments using a CrazyFlie 2.0 nano-drone equipped with a metal-oxide semiconductor (MOX) gas sensor. The authors found that the air drawn around the airframe strongly affected the sensor response, resulting in useless signals. They evaluated several mechanical solutions to keep the sensor out of the region of airflow created by the nano-drone, but the drone became uncontrollable because of inertia problems. The adopted solution was to operate the drone in the so-called “butterfly” mode, in which a human pilot lands the drone in the proximity of the source and halts the motors to take a measurement. In this way, the sensor signals are not affected by the rotors but, at the same time, the 3D sensing capabilities of the drone are not used, and the approach might not scale well to large scenarios. 

Fahad et al. [30] equipped the same nano-drone with a chemically sensitive field effect transistor (CS-FET) sensor for hydrogen (H_2_) detection. The test environment was a chemical hood in which H_2_ was released from the upper part of the hood and the drone ascended from the bottom of the hood to the area near the source (h = 60 cm), aided by high tension strings. The sensor response increased as the drone approached the source, reaching its maximum value after hovering (i.e., levitating) near the source for 40 s. The drone was merely used as a proof-of-concept demonstration of the proprietary gas sensor developed by the authors. The above works suggest that a nano-drone might be used for gas source localization (GSL), however the experimental scenarios were extremely simple.

### 1.2. Experimental Evaluation of Gas-Sensitive Nanodrones

The main problems for performing large-scale experiments in complex environments using nano-drones are related to the limited on-board resources and difficulty to control the platform due to inertia and stability issues. Taking as an example the CrazyFlie 2.0 (CF2) quadcopter, the tiny 240 mAh battery delivers power for up to 7 min of flight and 15 grams of payload, which means that only lightweight and power-efficient sensors can be mounted on board. Self-localization and obstacle avoidance—required for autonomous navigation—are hard to accomplish because laser scanners, for example, are too heavy for the nano-drone payload. Autonomous hovering of a CF2 equipped only with a tiny RGB camera has been achieved in indoor experiments [31], although the camera consumes all available payload and reduces the flight time to 3.5 min. The Global Positioning System (GPS) can be used for localization outdoors where, however, nano-drones can often not be controlled stably due to their low inertia and strong wind. Navigation in indoor areas can be achieved through motion capture systems (MOCAPs) [32] or radio frequency (RF)-based systems [33]. MOCAPs offer high accuracy (1 mm error) but are expensive, typically only cover small volumes and require line-of-sight (LOS) between the cameras and the drone. RF-based systems are cheaper, have a larger coverage area, do not necessarily require LOS but are less accurate (10 cm error). In many realistic scenarios, deploying an external localization system might not be possible (e.g., in a disaster situation) and the drone would have to navigate autonomously or remotely controlled by an operator.

Gas sensing tasks are also subject to additional constraints, as they must be executed in the short time limited by the battery capacity and relying exclusively on one or two chemical sensors. It should be noted that most research on GSL is based on terrestrial robots, which can be running for hours, perform long measurements of 1–2 min at each sampling location and possibly use selective sensors (e.g., TDLAS, OGI cameras, e-noses) and anemometers. Large drones can fly for 20–25 min and be equipped with the same technology as terrestrial robots. Nano-drones are therefore subject to unprecedented constraints because a stop-sense-go strategy would only allow for at most 10 measurements (of 30 s each), the limited number of sensors hinder rejecting chemical interferences and the absence of anemometry prevents assessing the wind direction, which is a key parameter for GSL. During its operation, the drone can also not fly too fast or the relevant structures of the chemical plume may become blurred due to the slow response time of the sensors [34,35]. Lilienthal et al. [35] pointed out that the gas distribution mapped by a terrestrial robot may be slightly shifted as compared to the real distribution, due to the memory effect of MOX sensors.

### 1.3. Gas Source Localization

**Figure 2:**
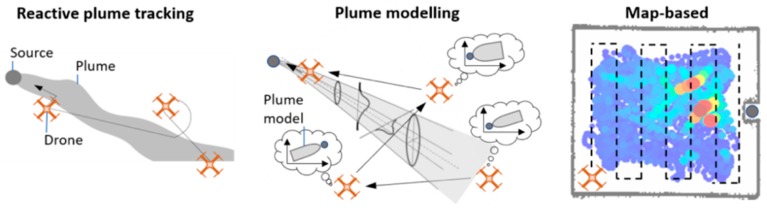
Gas source localization strategies. (**left**) Reactive plume tracking; (**center**) Plume modelling; (**right**) Map-based.

Gas source localization (GSL) is a key task for gas-sensitive robots that consists in identifying the point of release of a hazardous gas. GSL strategies can be divided into three groups: reactive plume tracking (bioinspired), plume modelling and gas distribution mapping (GDM) strategies [36] (Figure 2). Bioinspired algorithms attempt to track the gas plume along its entire length, mimicking the excellent odor plume tracking capabilities of some flying insects. At this time it is unclear whether bioinspired reactive behaviours have better performance than other approaches based on statistical inference from cumulative readings [37,38,39]. According to [11], the bioinspired reactive behaviors that researchers have implemented on mobile robots are modelled too simple to cope with complex environments, gas sensors used are too slow to resolve plume features in a milliseconds scale and mobile robots are not agile enough for performing insect-like reactive movements. Besides of that, bioinspired algorithms often require wind measurements (anemometry) and real-time obstacle detection, which hinders their use in nano-drones.

Plume modelling algorithms [40,41,42,43] assume a mathematical model for the plume, such as Gaussian shaped plumes [44] or filament/particle based models [45,46,47], and use local measurements of concentration and wind to fit the model and estimate the source location, which is usually a parameter of the model. The practical applicability of plume modelling methods is limited because they tend to make overly simplifying assumptions (e.g., that the wind field is stable, spatially uniform and measurable), often require a-priori information such as the source release rate in Gaussian models [40], or are sensitive to meta-parameters such as the odor detection threshold in filament-based models [41] or the probability of particle encounter as a function of distance to the source in particle models [42]. The Gaussian model also assumes that the exploration area does not contain obstacles or walls which could otherwise distort the plume. Further, long time-averaging might be required to observe a Gaussian plume [48,49] or to estimate the particle density in a certain region, which slows down plume modelling approaches for GSL.

GDM approaches use sensor measurements to first build a map of the gas distribution in the environment, which is then used to estimate the source location. Maps reflecting the instantaneous concentration [50], the mean concentration [35,38], the variance of the concentration [38,51] or the number of odor hits (which are over-threshold segments in the sensor response) [52] have been successfully used for GSL. To build a gas distribution map, the path of the robot should roughly cover the entire search area, typically moving along a predefined trajectory [35,38,50], although adaptative approaches have been proposed [53]. When the map is based on statistical properties of the gas distribution (e.g., mean or variance), long measurements (typically 30 s and more) are often carried out at each sampling location. This stop-and-sense strategy is not suitable for UAVs, particularly for nano-drones, since any hovering stop quickly drains the battery [15]. GDM algorithms are less efficient than bioinspired and plume modelling algorithms (in terms of distance travelled by the robot) but do not rely on unrealistic assumptions nor require wind information or a-priori parameters. They are compatible with slower robots and sensors and the resulting gas distribution map can be used for other purposes beyond GSL. 

MOX sensors are probably the most suitable gas sensing technology for nano-drones due to the reduced size (few mm^2^), low power consumption of some models (few mW) [54] and simplicity of the conditioning electronics. Lilienthal et al. [55] observed that the maximum response of a MOX sensor often corresponds to the approximate location of a gas source if the sensor readings are acquired in motion. Such a correlation was never observed if the concentration measurements were collected with a stop-sense-go strategy. This behaviour, which was previously reported by Atema et al. [56] and confirmed by Farah and Duckett [57], was attributed to the long recovery time of MOX sensors. If a MOX sensor is exposed to two consecutive gas patches, the response to the second stimulus will occur when the sensor has not yet recovered from the first exposure. The overall response to the second patch will be higher than if the sensor had been already fully recovered from the first patch. Since the local density of gas patches tends to be higher near the source, the encounter rate with gas patches is higher if the robot is moving. Thus, it is plausible that Rossi et al. [36] and Luo et al. [50] obtained a good estimate of source location in outdoor experiments using the instantaneous response of a MOX sensor mounted on a micro drone (≈800 g). In the latter case, the authors were able to build a 3D gas distribution map of a relatively large outdoor environment (10 × 16 m^2^) and localize the source in less than 10 min. For that, they used a predefined flight path consisting of two 2D rectangular sweepings at different heights (0.3 m and 1 m), without stopping at predefined locations for measuring. The cell with maximum value of the gas distribution map coincided approximately with the true source location. 

The results of Luo et al. [50] also show that the density of odor hits in a map is correlated to the source location. An odor hit is typically declared when the instantaneous concentration exceeds a certain threshold. Although the term ‘odor hit’ is widely used in the literature to indicate contact between the gas and the sensor, strictly speaking it would be more precise to refer to these events as ‘gas hits’ or ‘plume hits’ since most gases being released in reported experiments are odorless for humans. Nonetheless, Thomas Lochmatter [11] argues that the definition of odor as a gas that humans can smell can be extended to robots. In this sense, terms such as “odor” can also be used instead of “gas”. Odor hits are supposedly caused by contact with individual patches of the plume and there are indications that insects use similar features to orient rapidly in turbulent plumes [58,59]. Well-known GSL algorithms such as Pang and Farrell’s method [41] or Infotaxis [42] model the plume as a sequence of chemical filaments/particles and use odor hits to localize the source. Detecting odor hits with a MOX sensor is a challenging task due to the long recovery time (in the order of 10–30 s) compared to the temporal resolution of the chemical stimuli (in the order of ms). Several research groups attempted to improve the response time of MOX sensors by novel hardware designs [60,61] or by signal processing, using inverse dynamical models [34,62,63], artificial neural networks [64] or extracting specific features [65]. Hardware methods are not appropriate for nano-drones, as they usually increase the size, weight and power consumption of the system. Signal processing methods are more suitable. Schmuker et al. [65] proposed a method to extract short time-scale features (called ‘bouts’) from the derivative of the MOX sensor response that could be caused by contact with individual filaments of the plume. In wind tunnel experiments, the authors found that the frequency of these ‘bouts’ (as detected with MOX sensors) is strongly correlated to the distance of a gas source: the higher the bout frequency, the closer the sensor to the gas source. The proposed algorithm uses a threshold to filter out low-amplitude bouts—produced by sensor noise—that would otherwise lead to meaningless correlations. It was also found that the variance of the bout frequency (measured across multiple trials) indicates whether the detector is in the plume centerline (low variance) or slightly lateral from it (high variance). These features suggest a plume-tracking GSL strategy in which the robot first tries to locate the plume centerline by monitoring the variance of the bout frequency and then approaches the source by moving in the direction of increasing bout frequency. The advantages of this method are that anemometry is not required and, since bouts are detected in the derivative of the signal, the algorithm is not very sensitive to changes in the background concentration or to differences between individual gas sensors. The sensitivity of the algorithm to the threshold used to discard noise-induced bouts has not been studied yet, but it might have a large impact on the results. Since this method has not been experimentally validated beyond a wind tunnel, it remains to be shown if meaningful gradients of both bout frequency and its variance can be obtained in real scenarios. 

### 1.4. Proposed Smelling Nano Aerial Vehicle (SNAV)

In this work, we propose a Crazyflie 2.0 nano-drone equipped with a MOX sensor for gas source localization in large indoor environments. We calibrate the sensor to compensate the non-linear response, obtain measurements in concentration units and to estimate the limit of detection (LOD). We assess the impact of the propellers on the MOX sensor signals at different distances of a chemical source. We then compare two GSL strategies, one based on the instantaneous response and the other one based on the bout frequency in two experiments where the source is placed challenging positions for the drone. We show that proper selection of the bout amplitude threshold is critical for good localization performance. We also demonstrate that a 3D gas distribution map of an environment of 160 m^2^ can be built in less than 3 min using the proposed platform and the source can be accurately localized from the map.

## 2. Materials and Methods

### 2.1. Nano-Drone and Gas Sensors

Among all commercial nano-drones, we selected the CF2 (Bitcraze AB, Malmö, Sweden) due to its low cost, reduced dimensions (10 × 10 cm) and open hardware/software architecture. Weighing only 27 g, it has a maximum recommended payload of 15 g and is capable of up to 7 min of continuous flight. The main microcontroller (μC) is an ARM 32-bit STM32F405 Cortex-M4, which runs an open source real-time operating system (FreeRTOS). The CF2 communicates with a ground station (PC with USB radio antenna) over the 2.4 GHz ISM radio band in up to 1 km range line-of-sight (LOS). The CF2 periodically sends over this link internal variables of the system (sensor measurements, position, rotor speed, battery level, user-defined variables, etc.) and receives commands from the base station such as waypoints or parameter updates. An expansion port—accessible from top and bottom of the drone—provides the user with access to certain μC pins (4 x GPIO, 3 x PWM), power lines (GND, VCC 3.0 V, VBAT 3.7 V) and communication buses (I2C, SPI, 2 x UART). New hardware compatible with 3.0V logic can be easily integrated into the platform by soldering it into an expansion board, called a deck, which can be connected to the expansion port. An installed deck is automatically detected and initialized by the CF2 at startup, without having to modify the stock firmware. Only the deck driver needs to be programmed, a separate piece of code that specifies the functionality of the deck in FreeRTOS language.

**Figure 3:**
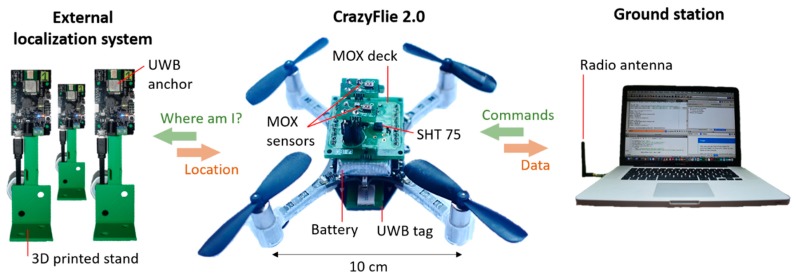
The CrazyFlie 2.0 equipped with the MOX deck and the UWB tag (**center**) gets its 3D position from an external localization system composed of six ultra-wide band anchors (**left**). The location and sensor data are communicated to the ground station (**right**) over the 2.4 GHz ISM band.

**Figure 4:**
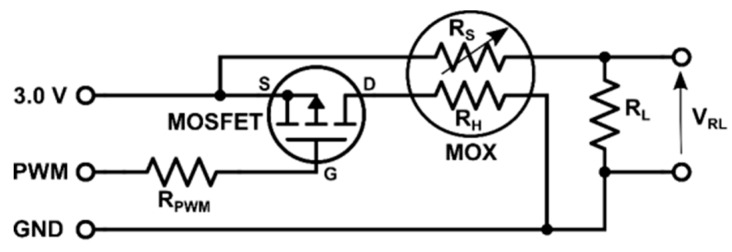
Schematic of the conditioning electronic circuit for each MOX sensor in the MOX deck, using PWM for powering and a voltage divider for read-out.

A custom deck (i.e., a printed circuit board), named the MOX deck, was developed to interface two MOX gas sensors to the CF2 (Figure 3). The deck contains two sockets for 4-pin Taguchi-type (TGS) gas sensors, a temperature/humidity sensor (SHT25, Sensirion AG, Stäfa, Switzerland), a dual-channel digital potentiometer (AD5242BRUZ1M, Analog Devices, Norwood, MA, USA) and two MOSFET p-type transistors (NX2301P, NEXPERIA, Nimega, NL). We selected the TGS 8100 sensor (Figaro Engineering Inc., Osaka, Japan) due to its compatibility with 3.0 V logic, power consumption of only 15 mW (the lowest in the market as of June 2016) and miniaturized form factor (MEMS). Since the sensor heater uses 1.8V, we included two transistors (one per sensor) to reduce the applied power by means of pulse width modulation (PWM). The MOX read-out circuit (Figure 4) is a voltage divider connected to the μC’s analog-to-digital converter (ADC). The voltage divider is powered at 3.0 V and the load resistor (R_L_) can be set dynamically by the potentiometer (from 60 Ω to 1 MΩ in steps of 3.9 kΩ). In the current work, we only used one of the two sensors and R_L_ was fixed to 70 kΩ. This load resistor value is selected to operate the voltage divider near its mid-range, where the sensitivity is maximum (according to the expected concentrations for the gas source described in Section 2.2).

The initialization task of the MOX deck driver configures the PWM, initializes the SHT25 sensor, sets the wiper position of both channels of the potentiometer and adds the MOX readout registers to the list of variables that are continuously logged and transmitted to the base station. The goal of PWM is to convert from 3.0 V to 1.8 V required by the MOX heater. For that, the PWM frequency is set to 8.4 KHz and the duty cycle (DC) to 36% according to

(1) $$DC=\frac{P_{\mathrm{avg}}}{P_{\mathrm{peak}}}=\frac{V_{\mathrm{avg}}^{2}/R}{V_{\mathrm{peak}}^{2}/R}=\frac{1.8^{2}}{3.0^{2}}=36\%,$$

where $P_{\mathrm{avg}}$ is the average power delivered to the sensor, $P_{\mathrm{peak}}$ is the peak power of the PWM signal and $P=V^{2}/R$ (Joule’s first law combined with Ohm’s law) is used to convert from power to voltage. The duty cycle is the fraction of time that the transistor delivers power to the sensor. 

The main task of the deck driver reads the MOX sensor output voltage and the temperature/humidity values from the SHT25 and sends them to the ground station at 10 Hz.

### 2.2. Experimental Arena, Gas Source and External Localization System

**Figure 5:**
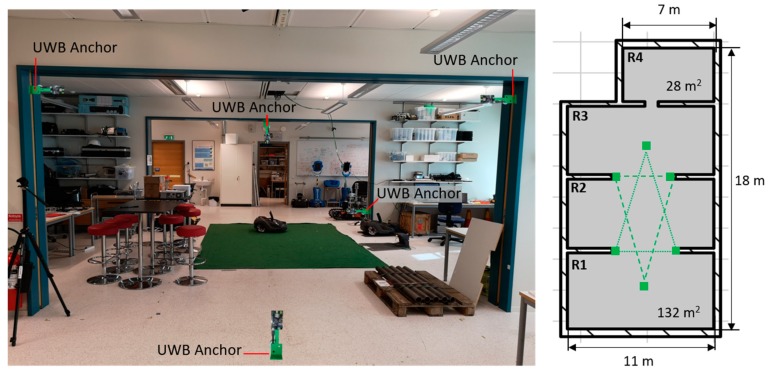
Experimental arena. (**left**) Frontal picture; (**right**) Schematic top view. The green squares indicate the position of the UWB anchors, which are positioned along two inverted triangles (green lines).

All experiments were performed in a large robotics laboratory (160 m^2^ × 2.7 m height) at Örebro University (Sweden). The laboratory is divided into three connected areas (R1–R3) of 132 m^2^ and a contiguous room (R4) of 28 m^2^ (Figure 5). The ventilation system of the laboratory was not modified for the experiments and all windows and doors were kept closed. 

To obtain the 3D position of the drone, we used an external localization system (Loco positioning system, Bitcraze AB) [66] based on ultra-wide band (UWB) radio transmitters. The system is composed of six anchors that are positioned in known locations of the room and one tag that is fixed to the drone. The anchors were placed in the central area of the laboratory, shaped in two inverted triangles (below and above the flight area), as recommended by the manufacturer. The tag on the drone continuously sends short high frequency radio messages to the anchors and estimates its relative position to them based on the timestamps of transmitted and received messages. The accuracy in the estimated position is approximately 10 cm if the tag is within the space delimited by the anchors and there is line-of-sight (LOS) between the anchors and the tag [67]. In the absence of these conditions, the system may still work but with degraded accuracy.

**Figure 6:**
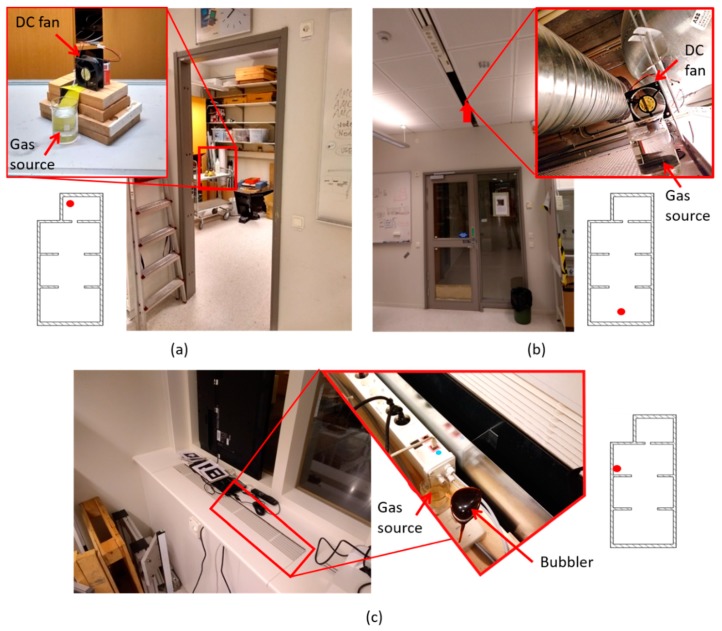
Gas source location in the three experiments. (**a**) Experiment 1: inside small room; (**b**) Experiment 2: hidden in suspended ceiling; (**c**) Experiment 3: hidden in a power outlet box.

A gas leak was emulated by placing a small beaker filled with 200 mL of ethanol 96% (Sigma–Aldrich, Germany) in different locations of the arena (Figure 6). Ethanol was used because it is non-toxic and easily detectable by MOX sensors. Three experiments were carried out to check the viability of the proposed system for GSL in complex environments. In the first experiment, the gas source was placed on top of a table (height = 1 m) in the small room (R4). In the second experiment, the source was placed inside the suspended ceiling (height = 2.7 m) near the entrance to the lab (R1). Since the piping system of the lab runs through the suspended ceiling, the gas source could represent a leak in one of the pipes. In these two experiments, a 12 V DC fan (Model: AD0612HB-A70GL, ADDA Corp., Taiwan) placed behind the beaker facilitated the dispersion of the chemicals in the environment, creating a plume. In the third experiment, the source was placed inside a power outlet box (height = 0.9 m) and a fish tank bubbler was used to increase the evaporation rate. The source in this location could simulate the early stages of an electrical fire (most of them are caused by faulty electrical outlets) where volatile organic compounds (VOCs) are released into the environment. The three experiments started five minutes after setting up the source and turning on the DC fan or the bubbler. 

### 2.3. Gas Sensor Calibration and Limit of Detection (LOD) Estimation

The MOX sensor was calibrated under laboratory conditions, to compensate the non-linear response and obtain measurements in concentration units. Ethanol concentrations up to 50 ppm were generated using the permeation method [68], humidified to 30 % r.h. and delivered in random order at 70 mL/min to the chamber containing the sensor under test. The uncertainty of the concentration reaching the gas chamber was determined by propagation of the main sources of error, namely the permeation rate, the oven temperature and the MFCs. Uncertainty values ranged from 50 ppb (at 1 ppm of ethanol) to 1 ppm (at 50 ppm of ethanol), which represents a relative uncertainty of 2–5%. The concentration range and humidity level for calibration samples were selected based on previous experience by the authors performing similar experiments in the same test environment. 

To estimate the LOD (ppm), we used the simplified LOD formula [69]:

(2) $$\mathrm{LOD}=\frac{3.3\times s_{0}}{\hat{A}},$$

where $s_{0}$ is the estimated standard deviation of blank measurements (assuming homoscedasticity and normality) and $\hat{A}$ is the estimated slope of the calibration graph (assuming linearity). To account for the expected variability between the calibration setup and the test environment [70], we estimated $s_{0}$ from a preliminary exploration of the target scenario in the absence of the gas. The LOD was used during the gas source localization experiments to remove false alarms, by setting to zero any measured concentration below the LOD.

### 2.4. Detection of ‘Bouts’

**Figure 7:**
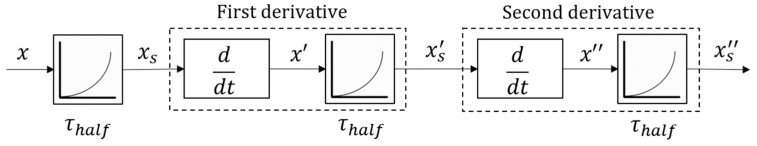
Flow diagram of the improved bout computation. The meaning of each symbol is given in the text.

To compute the ‘bouts’ from the MOX response, we adapted the signal processing pipeline proposed by Schmuker et al. [65]. The goal of this algorithm is to extract the rising edges of the smoothed derivative of the sensor response, which are called ‘bouts’. Schmuker’s algorithm is based on a non-causal Gaussian smoothing filter that prevents real-time bout detection, includes an unnecessary derivative, embeds the computation of the derivative within the smoothing filter (which may lead to potential implementation errors) and does not smooth the second derivative of the signal where the ‘bouts’ are segmented. The derivative that converts s to x in [65] is unnecessary because the $ema_{\alpha }$ transformation already differentiates the input signal. The source code published by Schmuker et al. is not affected by this error because the transformation is implemented by calling the Python function *pandas.ewma()*, which provides the functionality of an EWMA filter (i.e. it does not perform the derivative). We replaced the Gaussian smoothing filter with a causal (realizable) exponentially weighted moving average (EWMA) filter, removed the unnecessary derivative, decoupled the derivative from the EWMA filter and smoothed the second derivative. We chose the EWMA filter because it is causal, easy to implement and is the same filter used by Schmuker et al. to smooth the derivative in their bout computation algorithm. The proposed bout computation pipeline is represented in Figure 7. 

The sensor response x is first smoothed using a EWMA low-pass filter to remove high-frequency noise. At time t, the smoothed value $x_{s}\left(t\right)$ is found by computing

(3) $$x_{s}\left(t\right)=\left(1-\alpha \right)\cdot x_{s}\left(t-1\right)+\alpha \cdot x\left(t\right),$$

where x(t) is the observation at time t, $x_{s}\left(t-1\right)$ is the previous output of the filter and the smoothing factor α (0 < *α* ≤ 1) controls the speed at which older responses are dampened. The smoothing factor *α* in the EWMA filter is equivalent to the cut-off frequency of a low-pass filter. For example, Pashami et al. [71] found out that *α* = 0.9 represents a cut-off frequency of 0.44 Hz. It is convenient to express the smoothing factor as a function of the half-life time $\tau_{\mathrm{half}}$ (s)

(4) $$\alpha =1-\exp\left(\frac{\log0.5}{\tau_{half}\cdot f_{s}}\right),$$

where $f_{s}$ is the sampling frequency (Hz) of x. The smoothed response is differentiated and smoothed again to increase the signal-to-noise ratio (SNR), producing $x_{s}^{\prime }$. The ‘bouts’ are the rising edges of $x_{s}^{\prime }$, which are delimited by two consecutive zero-crossings in the positive derivative of $x_{s}^{\prime }$, i.e., $x_{s}^{\prime \prime }>0$. The amplitude of a bout is defined as $x_{s}^{\prime }$ at the end of the respective bout segment minus $x_{s}^{\prime }$ at the start of the same bout segment. To remove low-amplitude bouts produced by noise, Schmuker et al. propose to estimate the noise threshold ($b_{thr}$) using the 3-sigma rule

(5) $$b_{thr}=\mu +3\sigma ,$$

where *μ* and *σ* are the estimated mean and standard deviation, respectively, of the distribution of amplitudes of bouts detected in the sensor baseline (i.e., in the absence of gas). Bouts with amplitude lower than $b_{thr}$ are filtered out. The algorithm that we propose reduces the number of parameters from three to two ($\tau_{\mathrm{half}}$ and $b_{thr}$), which we estimate after a preliminary exploration of the target scenario in the absence of gas (i.e., using the signals corresponding to blank measurements).

### 2.5. Effect of Rotors on MOX Sensor Signals

**Figure 8:**
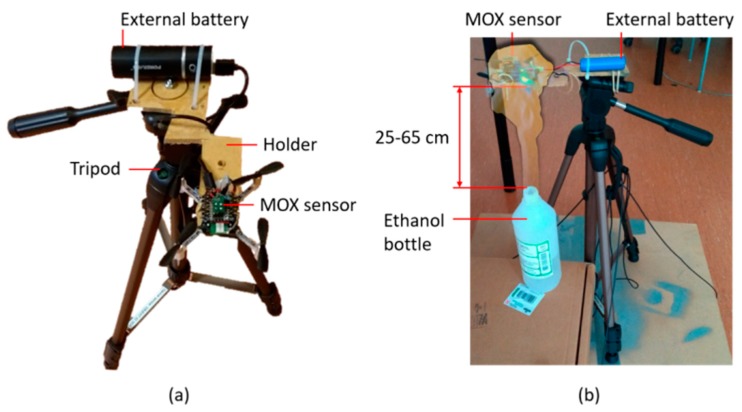
Setup for assessing the effect of the rotors on the MOX sensor signals. (**a**) Top view of the stand used to hold the drone at different heights while minimizing interference with the rotors air flow; (**b**) Photo of an experiment with the drone placed 25 cm above an ethanol bottle (gas source), overlaid with an illustration of a gas cloud.

Previous work using the CF2 indicate that turbulence generated by the propellers may severely affect the MOX sensor signals [29]. To evaluate this effect in our platform, we performed a set of measurements near a gas source under two conditions: rotors switched on or rotors switched off. The drone was placed on a height-adjustable stand—designed to minimize its interference with the rotors’ airflow—that could be moved around the source (Figure 8a). Using a stand is necessary to perform measurements when the rotors are switched off, and we also used it to perform measurements with the rotors switched on. The gas source was an open ethanol bottle (Figure 8b) and measurements were performed above the source (vertical distance between 25 and 65 cm) and in front of it (at 50 cm) At each location, the sensor response was recorded for 25 min (using an external battery), first with the rotors switched off and, after cleaning the room, measurements were repeated with the four rotors spinning at 10,000 rpm (this is typical for hovering). 

### 2.6. Gas Source Localization Strategies

**Figure 9:**
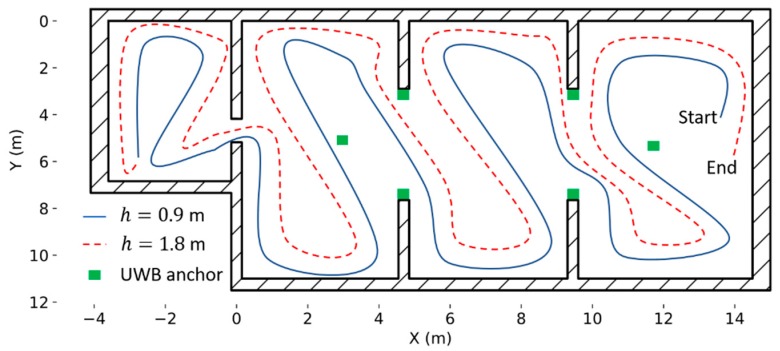
Predefined navigation strategy based on zig-zag sweeping at two heights (0.9 and 1.8 m). The green squares indicate the location of the UWB anchors.

Two gas source localization strategies, one based on the instantaneous response and the other one based on odor hits are evaluated using the nano-drone. In both cases, the drone was sent to fly along a predefined sweeping path consisting of two 2D rectangular sweepings at different heights (0.9 m and 1.8 m), collecting measurements in motion (Figure 9). These two heights divide the vertical space of the lab in three parts of equal size. Flying first at a lower altitude minimizes the impact of the propellers’ downwash in the gas distribution. For safety reasons, the trajectory is designed to ensure enough clearance around obstacles and walls, and people working inside the laboratory were told to remain in their seats during the experiments. The ground station communicates the flight path to the drone as a sequence of (x,y,z) waypoints, with a target flight speed of 1.0 m/s.

As the drone navigates the environment, it reports the instantaneous concentration and its location to the ground station. At the end of the exploration, the ground station uses all the received information to compute a 3D map of the instantaneous response (first strategy) and the bout frequency (second strategy). The location of the gas source is estimated in both cases as the cell of the map with maximum value. We will also discuss the viability of both methods for real-time plume tracking, assuming that the drone would follow the gradient of instantaneous concentration or the gradient of bout frequency (computed using a sliding window of 5 s).

## 3. Results

### 3.1. Calibration, LOD and Optimum Parameters for Bout Detection

**Figure 10:**
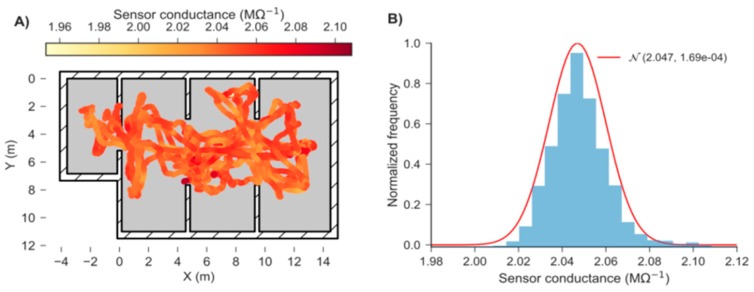
(**A**) 2D map of MOX sensor response during 15 min of random exploration of the target area without gas; (**B**) Histogram of blank readings, with a Gaussian curve *Ν*(μ, $\sigma^{2}$) superimposed.

**Figure 11:**
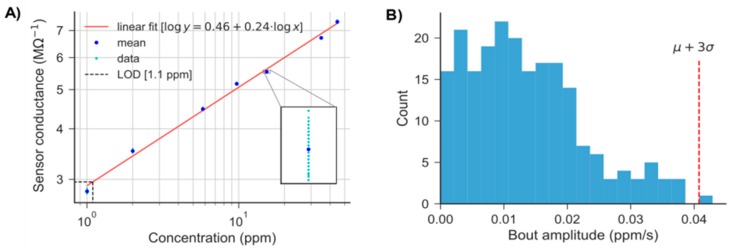
(**A**) Calibration line in the range 1–50 ppm (log-log plot), with blank variability superimposed at each concentration level (see inset). The LOD is estimated using Equation (2); (**B**) Histogram of amplitudes of bouts detected in the calibrated blank signals. $b_{thr}$ (Equation (5)) is indicated by a red dashed vertical line.

A preliminary exploration of the test environment in the absence of gas was performed to estimate the sensor noise and the optimum bout parameters (Figure 10A). The raw response was smoothed using an EWMA filter with a smoothing factor $\tau_{\mathrm{half}}=0.25\;s$. The noise can be approximated by a Gaussian distribution with mean value of $2.047\;M{Ω}^{-1}$ and standard deviation of $0.013\;M{Ω}^{-1}$ (Figure 10B). The observed variability was used in combination with the calibration line (Figure 11A) to estimate the LOD (Equation (2)), assuming homoscedasticity. The calibration line behaved linearly in the range 1–50 ppm after applying the logarithm to both concentration and response. The bout amplitude threshold ($b_{thr}$) was estimated as 0.04 ppm/s by applying Equation (5) to the amplitude of the bouts detected in the calibrated blank signals (Figure 11B).

### 3.2. Effect of Propulsion on MOX Signals

**Figure 12:**
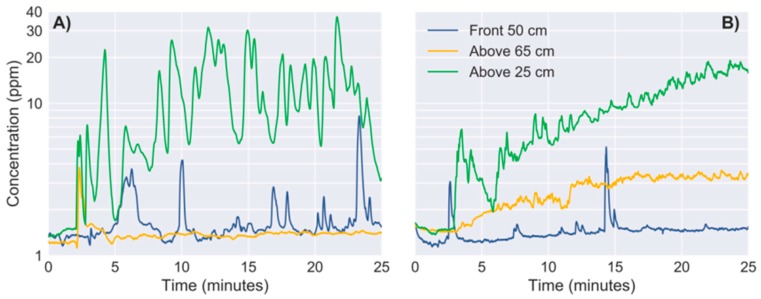
Sensor signals (log scale) near an evaporating source. (**A**) Propellers switched off; (**B**) Propellers switched on. The ethanol bottle is opened at t = 2 min.

**Figure 13:**
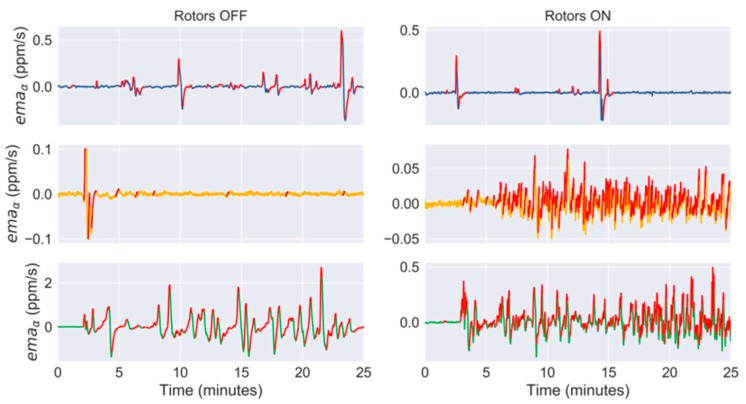
Smoothed derivative (i.e., $x_{s}^{\prime }$ in Figure 7) of the sensor signals at 50 cm in front of the source (blue line), 65 cm above the source (yellow line) and 25 cm above the source (green line). Bouts with amplitude higher than μ+3σ are highlighted in red. In the left column, the propellers are switched off whereas in the right column they are switched on. The ethanol bottle is opened at t=2 min.

When the propellers were switched off (Figure 12A), the concentration fluctuations due to the gas evaporating from the ethanol bottle were clearly reflected on the on-board sensor signals at 25 cm above the source (green trace) and, to less extent, at 50 cm in front of the source and (blue trace). At 65 cm above the source (yellow trace), the sensor does not seem to detect the source except immediately after opening the bottle (t=2 min). When the same measurements were repeated with the propellers switched on (Figure 12B), the fluctuations of the signals at 25 cm above the source became less intense but more frequent. This can be better observed in Figure 13, where the bouts detected with the propellers switched on are as twice as frequent than when the propellers are switched off (Table 1). This effect is even more noticeable at 65 cm above the source because the bout frequency increases from 0.48 to 7.74 bouts/min by switching on the propellers, improving the detection of the source. The propellers produced nonetheless a negative effect in the signals acquired in front of the source, reducing the bout frequency by a factor of two. 

**Figure 14:**
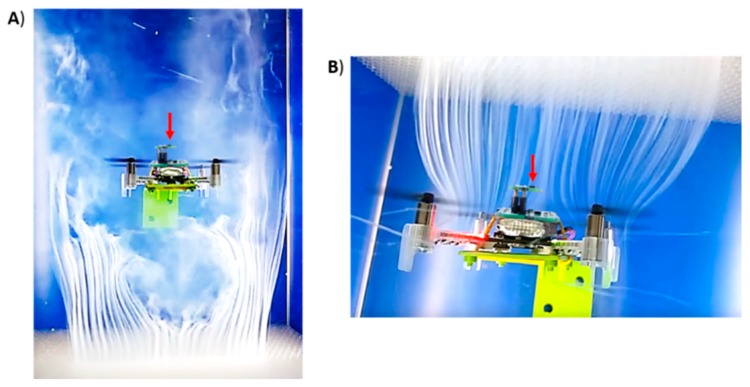
Aerodynamics of Crazyflie 2.0 when the four rotors are spinning, visualized using a Deskbreeze wind tunnel (Courtesy of Bitcraze AB). The drone is fixed to one of the walls of the tunnel using a 3D printed stand and dry ice fog is emitted from (**A**) below the drone or (**B**) above the drone. It shows the downwash of the propellers and how part of the fog reaches the MOX gas sensor (red arrow). The MOX deck has been overlaid to the original images for visual clarity.

The steady increase of the average concentration and higher bout frequency observed when the drone was sampling above the source with the rotors switched on can be explained based on how the propellers interact with the gas surrounding the drone. The propellers generate a downwash (i.e., a downward airflow) that acts as an opposing force to the gas moving upwards by convection, breaking the laminar flow into a turbulent gas cloud that spreads around the drone’s frame (Figure 14A), probably increasing the background concentration. When the gas is spread around the drone, the propellers drag the gas patches towards the MOX sensor (Figure 14B) and this probably increases the bout frequency. Therefore, from this study we can conclude that the propellers severely change the gas distribution near the drone, which in turn affects the MOX signals. However, it may still be possible to extract relevant features for gas source localization (e.g., the bout frequency).

### 3.3. Experiment 1: Localization of a Source 17 m Away from the Starting Point

**Figure 15:**
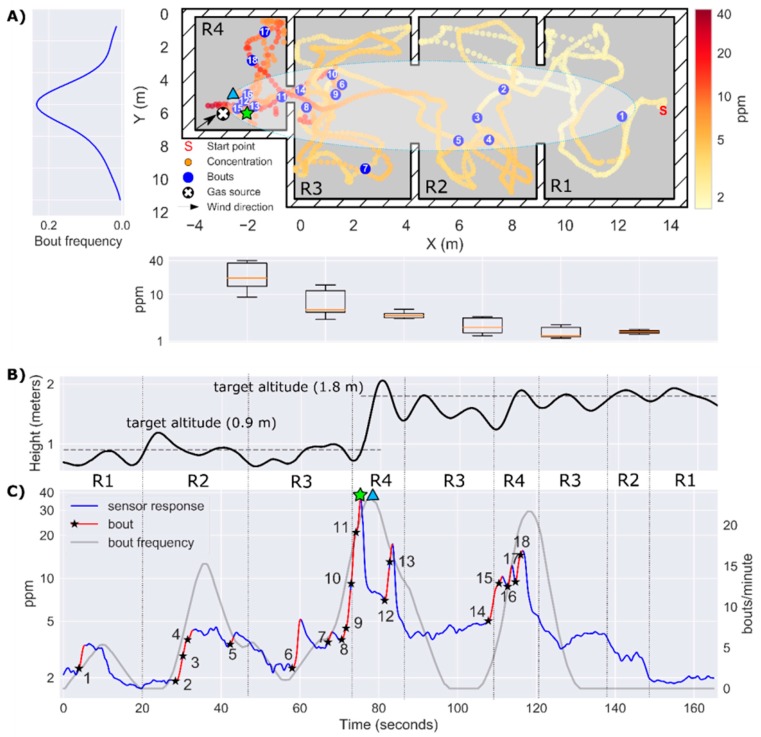
Results of Experiment 1. (**A**) 2D map of the instantaneous concentration (ppm) in log scale, with bouts represented by blue circles ($b_{thr}=0.52$ ppm/s). A hand-drawn ellipse outlines the approximate plume shape based on the location of bouts. The average bout frequency along the y-axis is shown in the leftmost panel. The box plot below the map represents the instantaneous concentration along the x-axis; (**B**) Trajectory of the drone along the z-axis. (**C**) Temporal evolution of the instantaneous concentration (ppm) on a log scale, with detected bouts highlighted in red (the black star indicates the start of a bout). The identifiers R1–R4 between panels (**B**) and (**C**) indicate the area of the map in which the drone is flying at each moment. The maximum instantaneous concentration and the maximum bout frequency are indicated by a green star and a blue triangle, respectively.

In the first experiment, the drone took off near the entrance of the lab (R1), 17 meters downwind of a gas source located in the other end of the laboratory (R4). Flying along a predefined trajectory that took 2.75 min, the drone acquired measurements to build a map of the instantaneous response (Figure 15A). From this map it is evident that the gas source must be in R4, because the maximum concentration (35 ppm) was found there while concentrations below 5 ppm were measured in the rest of the lab. The gas plume can be outlined from the obtained bout map, which shows the highest bout density (25 bouts/min) also in R4. Most bouts were detected during the first part of the exploration (Figure 15C), when the drone was flying at the same height of the source (Figure 15B). Nonetheless, multiple bouts were also detected flying above the source in R4 (t= 110–120 s). The cells corresponding to the maximum concentration and maximum bout frequency were found at 0.94 and 1.16 m of the true source location, respectively.

For real-time plume tracking, following the gradient of instantaneous concentration would not easily lead the drone towards the source, since the concentrations measured in R1 are only slightly higher than the LOD of the sensor (1.1 ppm). Although it is true that the instantaneous concentration increases when the drone crosses the plume (e.g., bouts #1, #2 and #6), it is not clear when the drone exits the plume due to the increase in background concentration and slow sensor recovery. In this sense, the bout frequency behaves like a more sensitive version of the instant response, exhibiting abrupt changes when the drone enters/exits the plume. This can be seen at t=85 s, when the drone exits R4 and enters R3. The bout frequency sharply drops to zero, but the instantaneous concentration remains flat at 5 ppm. Another example is found by comparing both features when the drone crosses the door that connects R3 to R4 flying at h=0.9 m (t=75 s) or at h=1.8 m (t=110 s). In the first case, the instantaneous concentration jumps from 4 to 35 ppm whereas in the latter case the step is only from 5 to 10 ppm. On the other hand, the bout frequency shows a similar increase (0 to 23–25 bouts/min) in both cases. This could mean that the bout frequency, which simply counts events regardless of their amplitude is more robust for detecting the source when its height is unknown. 

**Figure 16:**
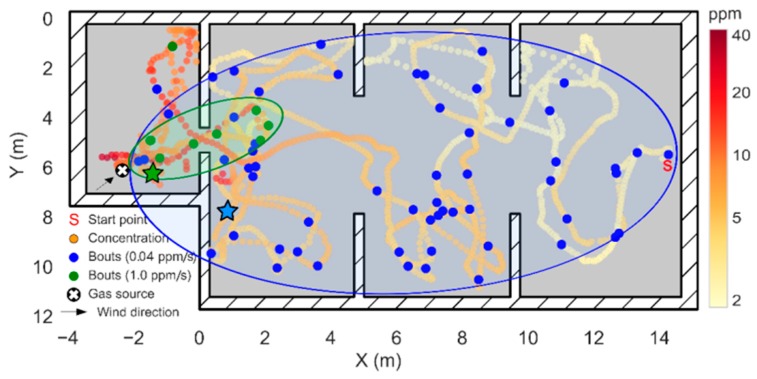
Effect of the bout amplitude threshold in the results of Experiment 1. The blue circles represent bouts with amplitude higher than 0.04 ppm/s (μ+3σ threshold) and the green circles represent bouts with amplitude higher than 1.0 ppm/s. In each case, a hand-drawn ellipse outlines the approximate plume shape based on the location of the bouts. The green and blue stars indicate the source location estimate in each case, according to the maximum bout frequency.

To obtain the bout map shown in Figure 15A, we had to increase the bout threshold ($b_{thr}$) from 0.04 ppm/s (computed using Equation (5)) to 0.52 ppm/s (determined from visual inspection). The effect of the bout amplitude threshold on the bout map is show in Figure 16. If the threshold is set too low (blue circles), slight variations of the background concentration can trigger bout detections that are not necessarily produced by the gas plume, resulting in a sparse distribution of bouts that hinders the localization of the source (localization error of 4.32 m). This variability in the background concentration may be caused by the downwash of the drone, which breaks the plume and disperses fragments of the plume in the room. On the other hand, if the threshold is set too high (green circles) it is virtually guaranteed that every detected bout is produced by the plume. However, in this case, bouts appear only near the source, which reduces the distance at which the source can be detected. Only when the threshold is suitably estimated (Figure 15A), the bout map resembles an elongated plume that can be used to move towards the source from a long distance. As of now, we are not aware of a systematic approach to properly set this threshold without prior information about the source intensity. One idea could be to use a multi-threshold approach, i.e., building bout maps with different thresholds and searching for consistent source predictions over multiple threshold values.

### 3.4. Experiment 2: Localization of a Source Hidden in the Suspended Ceiling (h = 2.7 m)

**Figure 17:**
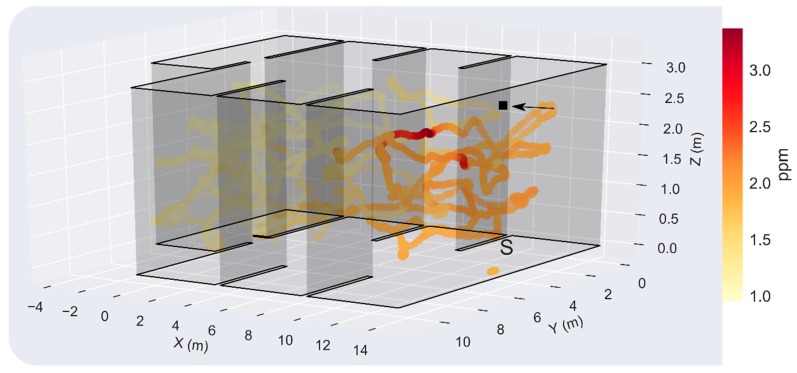
3D map of the instantaneous concentration (ppm) in Experiment 2. The black square indicates the gas source location (*x,y,z*) = (14.0, 5.2, 2.7) m, the black arrow the wind direction (positive x-axis) and the letter ‘S’ the starting point of the drone (*x,y,z*) = (13.5, 5.2, 0.0) m.

**Figure 18:**
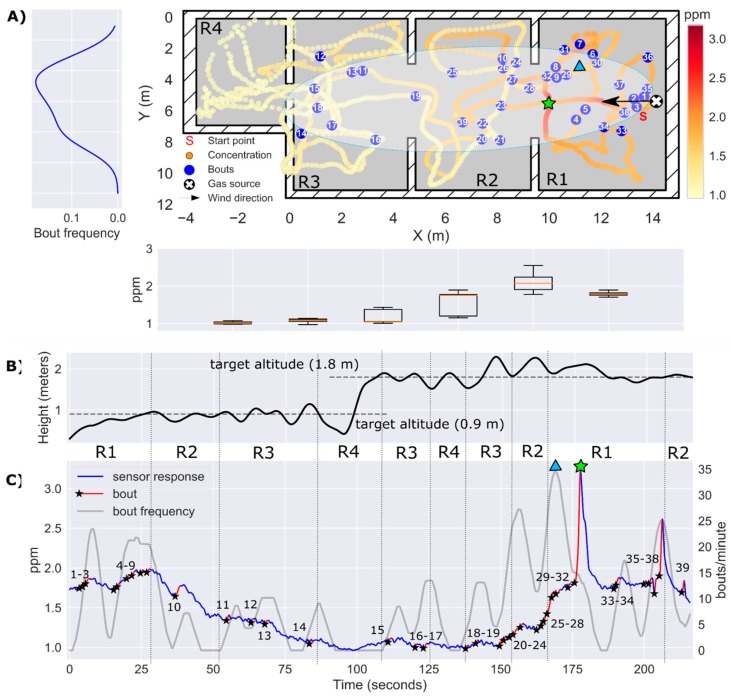
Results of Experiment 2. (**A**) 2D map of the instantaneous concentration (ppm), with odor hits represented by blue circles ($b_{thr}=0.04$ ppm/s). A hand-drawn ellipse outlines the approximate plume shape based on the location of bouts. The average bout frequency along the y-axis is shown in the panel on the left. The box plots below the map represents the instantaneous concentration along the x-axis; (**B**) Drone trajectory in the z-axis. (**C**) Temporal evolution of the instantaneous concentration (ppm), with detected bouts highlighted in red (the black star indicates the start of the bout). The bout frequency (gray line) is computed using a sliding window of 5 s. The identifiers R1–R4 between panels (**B**) and (**C**) indicate the area of the map in which the drone is flying at each moment. The maximum instantaneous concentration and the maximum bout frequency are indicated by a green star and a blue triangle, respectively.

**Figure 19:**
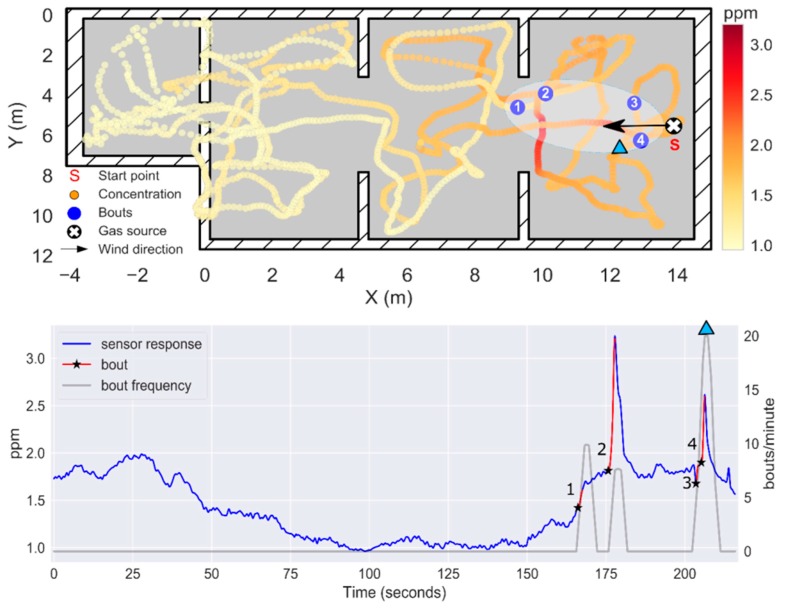
Results of Experiment 2 when $b_{thr}$ is increased to 0.18 ppm/s. (**top**) 2D map of the instantaneous concentration (ppm), with odor hits represented by blue circles. A hand-drawn ellipse outlines the approximate plume shape based on the location of bouts. (**bottom**) Temporal evolution of the instantaneous concentration (ppm), with detected bouts highlighted in red (the black star indicates the start of the bout). The bout frequency (gray line) is computed using a sliding window of 5 s. The maximum bout frequency is indicated by a blue triangle.

In this experiment, the source was located just above the starting point of the exploration, hidden in the suspended ceiling (Figure 17). The resulting maximum concentration in the test room was measured when the drone flew at h=1.8 m, highlighting the importance of sampling in 3D for localization and mapping of elevated gas sources. However, since the source is presumably not directly exposed to the environment, concentrations below 3 ppm were found in most locations of the room, which complicates the GSL task. The μ+3σ threshold yielded better results than in the previous experiment, outlining a feasible plume shape according to the true gas source location and predominant wind direction (Figure 18A). The instantaneous response map and the bouts map suggest that the gas source is in the division between R1 and R2, which represents a localization error of 4.0 and 3.31 m, respectively. The localization error can be reduced to 2.22 m by increasing the bout threshold from 0.04 ppm/s to 0.18 ppm/s (Figure 19). In this case, the lower inter-bout interval between bouts #3 and #4 (as compared to bouts #1 and #2) leads to a higher bout frequency near the source.

### 3.5. Experiment 3: Localization of a Source Hidden Inside a Power Outlet Box (h = 0.9 m)

**Figure 20:**
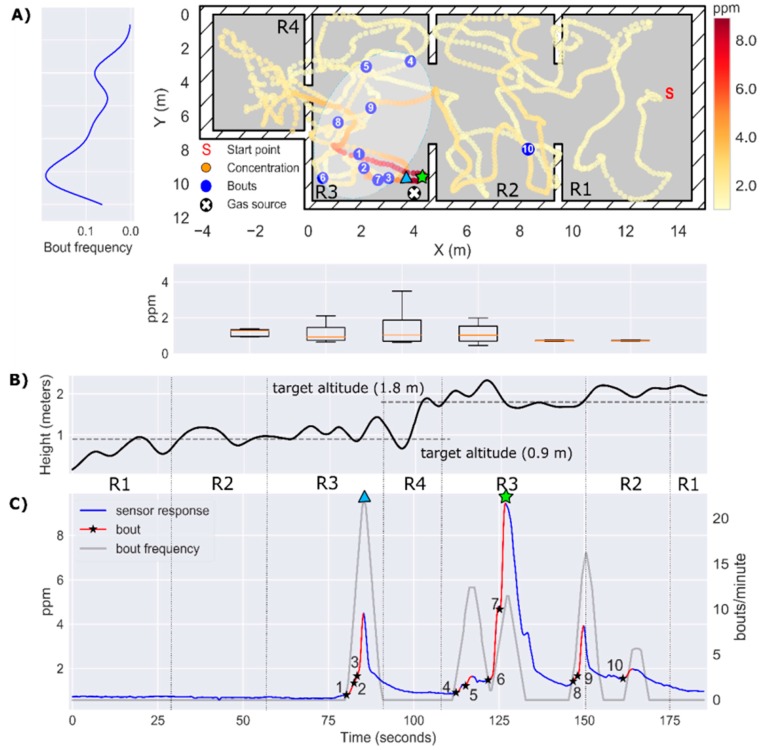
Results of Experiment 3. (**A**) 2D map of the instantaneous concentration (ppm), with bouts represented by blue circles ($b_{thr}=0.20$ ppm/s). A hand-drawn ellipse outlines the approximate plume shape based on the location of bouts. The average bout frequency along the y-axis is shown in the leftmost panel. The box plot below the map represents the instantaneous concentration along the x-axis; (**B**) Drone trajectory in the z-axis. (**C**) Temporal evolution of the instantaneous concentration (ppm), with detected bouts highlighted in red (the black star indicates the start of the bout). The bout frequency (gray line) is computed using a sliding window of 5 s. The identifiers R1–R4 between panels (**B**) and (**C**) indicate the area of the map in which the drone is flying at each moment. The maximum of the instantaneous concentration and the bout frequency are indicated by a green star and a blue triangle, respectively.

In this experiment, the gas source was placed inside a power outlet box and a bubbler was used to increase the release rate (no artificial airflow). The gas distribution map (Figure 20A) shows a local accumulation of gas in R3 near the source (max concentration = 8 ppm), whereas in the rest of the map the gas concentration is below 1 ppm. The bout map does not resemble the elongated plume observed in previous experiments probably due to the absence of induced airflow. During the first part of the exploration at h=0.9 m, the measured concentration was constantly below the LOD and no bouts were detected (Figure 20C), which represents a clear challenge for a robot attempting to reactively track the plume. Only when the drone approached the source (d<3 m) the first bouts were detected (bouts #1–3). The remaining bouts and the maximum concentration were found during the second pass at h=1.8 m, which may indicate a buoyant gas dispersion due to convective air currents produced by heat inside the power outlet box. The location of the source in the instantaneous concentration map and using the bout frequency were very accurate in the x-y plane (error of 0.7 m), but the bout frequency yielded a lower localization error (0.77 m) than the instantaneous response (1.22 m) because the estimated height of the source in the first case (1.2 m) was closer to the true value (0.9 m) than in the second case (1.7 m).

### 3.6. Overall Localization Results

A summary of the localization results is given in Table 2. The bout frequency with optimum noise threshold achieved comparable results to the instantaneous concentration, except in Experiment 2 where the former achieved better performance (2.2 m versus 4.0 m error). The bout frequency ((μ+3σ) threshold) produced relatively high localization errors.

## 4. Discussion

Our results suggest that a gas-sensitive NAV can be used for gas source localization and mapping in large indoor environments. The two GSL strategies compared in this paper require either building a map of the gas distribution or a map of the bouts. In contrast to previous works in which long measurement times are needed at predefined or adaptively chosen sampling locations, we have demonstrated that a rough approximation of such maps can be obtained in very short time with concentration measurements acquired in motion. Both maps seem coherent with respect to the true source location and wind direction, and not only enable the detection of the source with relatively small localization errors but also provide a rich visual interpretation of the gas distribution, especially if the bouts are overlaid on the gas distribution map. We adapted the bout detection algorithm for real-time operation and optimized the noise threshold, discussing how it affects the results in each experiment. The instantaneous gas distribution provides a more intuitive representation of the gas source location whereas the bout map can outline the gas plume without requiring anemometry.

The experiments presented in this paper also demonstrate that the air flow generated by the propellers greatly affects the gas sensor signals, but key information for GSL can still be extracted by signal processing methods. This appears to contradict the results reported by Rossi et al. [29] who claimed that the signals of a MOX sensor on board of a Crazyflie 2.0 are useless if the propellers are switched on. It is difficult to reason about the causes of this discrepancy because Rossi et al. do not show nor describe the experimental arena in which their experiments were performed, including the type of gas source and its location relative to the drone position. If they attempted to measure the gas coming out from an evaporating chemical source placed right below the drone, we have seen in this work that the downwash of the drone prevents most of the gas reaching the sensor surface and this produces raw signals with much less fluctuations than they would have if the same measurements were carried out with the propellers turned off (see green trace in Figure 12). We have also seen that such characteristic fluctuations can be still extracted from the smoothed derivative of the response (Figure 13). Another possible reason for this difference may be that different MOX sensors were used (MiCS 5525 for CO detection in [29] and Figaro TGS 8100 in this work) or they were operated differently. While we operate the sensor in continuous mode, Rossi et al. apply duty-cycling to the sensor heater to reduce the power consumption, which is known to degrade the signal quality [54]. Other reasons may be the signal processing carried out, the test gas concentrations or the range of variation of environmental conditions. If the gas concentrations are close to the LOD of the sensor, any turbulence created by the propellers or slight changes in ambient temperature or humidity may hinder distinguishing the response to the gas from the noise [72].

We dedicated a substantial portion of the effort to calibrate the MOX sensor in similar conditions to the test scenario and to find a suitable linear calibration model that enables the computation of the LOD using the standard equations. The log-log transformation linearized the MOX response in the range 0–50 ppm and the LOD was estimated as 1.1 ppm. This is something not usually done in the field of mobile robot olfaction since calibration of gas sensors is time-consuming and requires expensive dedicated equipment. The sensor signals are often uncalibrated [73,74,75,76] or only scaled to unit range [77,78,79,80], which produces maps that represent the sensor response instead of the absolute concentration. Response maps are not easily interpretable due to the non-linear sensor response and are sensor-dependent; i.e., two response maps obtained with different gas sensors may show notable differences even if the underlying gas distribution is similar. Scaling is a linear operation that cannot remove non-linearities of the original signals. Some authors assume a linear response within a certain concentration range [75,76]; however, for most gas sensing technologies (e.g., MOX, gasFETs or thermoelectric sensors) this is only true within a narrow range of concentrations (e.g., 0–10 ppm) often exceeded by the typical concentrations obtained in real experiments.

Most GSL algorithms require a detection threshold that is used to differentiate when a given sensor reading is produced by the gas plume or by background noise. Algorithms based on ‘odor hits’ require such a threshold to binarize the sensor response or to filter out low-amplitude bouts produced by noise. The rapid decay in chemical concentration with increasing distance from the source combined with incorrectly estimated detection thresholds may lead to a reduced area where the plume can effectively be detected (if the threshold is too high) or a high number of false alarms (if the threshold is too low). This has not been reported in the literature as an issue in GSL experiments because the concentration was often unrealistically high and the arena too small, i.e., the robot was very close to the source during the whole experiment. In this work, we challenged the drone by placing the source up to 17 m from the starting point or hiding it in the suspended ceiling or inside a power outlet box, leading to concentrations near the LOD of the sensor. It seems that for proper estimation of the noise threshold it is critical to have some prior information regarding the source intensity. A threshold computed exclusively from measurements in the absence of gas (e.g., μ+3σ threshold) may only work when the concentrations in the room are small. If the background concentration in the room increases, for example due to gas dispersed by the propellers of the drone, the threshold must be increased to reduce false bout detections. This suggests a multi-threshold approach, i.e., building bout maps with different thresholds and searching for consistent source predictions over multiple threshold values.

An obvious future direction for nano-drones is to achieve autonomous navigation, i.e., without resorting to external localization systems. This requires three-dimensional obstacle detection and self-localization, which is not an easy task whatsoever because there are many degrees of freedom and the low payload does often not allow for range sensors. In this line, the Fast Lightweight Autonomy (FLA) program (https://www.darpa.mil/program/fast-lightweight-autonomy) from the Defense Advanced Research Projects Agency (DARPA) is exploring novel perception methods and algorithms for small autonomous UAVs to fly at speeds up to 20 m/s in cluttered environments with no remote pilot, no communication links, no GPS guidance and no pre-programmed map of the area. In phase 2 of the program (June 2018), successful tests were performed with drones weighing around 2 kg equipped with a single camera, flying through a narrow window into a building, searching rooms, creating a 3-D map of the interior and exiting the building through an open doorway. In this paper we have demonstrated that a gas sensor could complement the camera in this application, adding a gas distribution layer to the acquired map of the environment which could be eventually analyzed by an operator to identify potential leak sources or victims.

Before reaching this goal, the SNAV shall be tested in more complex scenarios, for example in environments with chemical interferences or multiple gas sources. In this context, it may be required to use multiple MOX sensors, integrate air flow information or implement more advanced localization/mapping algorithms, for example bioinspired reactive plume tracking or BASED maps of bouts [81,82] (i.e., using bouts as events or as weighted events).

## Figures and Tables

**Table 1:** Characterization of MOX signals at different distances of the source under two conditions: propellers switched on or off.

Distance	Propellers	Mean (ppm)	Variance (ppm^2^)	Bout Frequency (Bouts/min)	Bout Amplitude (ppm/s)
Above 25 cm	OFF	10.05	60.46	3.52	0.39
	ON	9.22	29.97	7.69	0.084
Above 65 cm	OFF	1.39	0.053	0.48	0.027
	ON	2.67	0.53	7.74	0.015
Front 50 cm	OFF	1.68	0.59	1.13	0.10
	ON	1.45	0.12	0.47	0.10

**Table 2:** Gas source localization error (m) in the three experiments, using the instantaneous concentration, the bout frequency with μ+3σ threshold or the bout frequency with optimum threshold.

Experiment	Instantaneous Concentration	Bout Frequency (μ+3σ)	Bout Frequency (Optimum Threshold)
1	0.94	4.32	1.16
2	4.0	3.31	2.22
3	1.22	5.07	0.77
Mean	2.05	4.23	1.38
